# Effect of Loquat Leaf Extract on Muscle Strength, Muscle Mass, and Muscle Function in Healthy Adults: A Randomized, Double-Blinded, and Placebo-Controlled Trial

**DOI:** 10.1155/2016/4301621

**Published:** 2016-11-24

**Authors:** Young Hye Cho, Sang Yeoup Lee, Cheol Min Kim, Nam Deuk Kim, Sangmin Choe, Chang-Hyung Lee, Jin-Hong Shin

**Affiliations:** ^1^Family Medicine Clinic, Obesity, Metabolism and Nutrition Center and Research Institute of Convergence of Biomedical Science and Technology, Pusan National University Yangsan Hospital, Yangsan, Republic of Korea; ^2^Department of Medical Education, Pusan National University School of Medicine, Yangsan, Republic of Korea; ^3^Center for Anti-Aging Industry, Pusan National University, Busan, Republic of Korea; ^4^Department of Biomedical Informatics, Pusan National University School of Medicine, Yangsan, Republic of Korea; ^5^Department of Pharmacy, Molecular Inflammation Research Center for Aging Intervention, Pusan National University, Busan, Republic of Korea; ^6^Department of Clinical Pharmacology and Therapeutics, Pusan National University Hospital, Busan, Republic of Korea; ^7^Department of Rehabilitation Medicine, Pusan National University Yangsan Hospital, Yangsan, Republic of Korea; ^8^Department of Neurology, Pusan National University Yangsan Hospital, Yangsan, Republic of Korea

## Abstract

Ursolic acid (UA) is the major active component of the loquat leaf extract (LLE) and several previous studies have indicated that UA may have the ability to prevent skeletal muscle atrophy. Therefore, we conducted a randomized, double-blind, and placebo-controlled study to investigate the effects of the LLE on muscle strength, muscle mass, muscle function, and metabolic markers in healthy adults; the safety of the compound was also evaluated. We examined the peak torque/body weight at 60°/s knee extension, handgrip strength, skeletal muscle mass, physical performance, and metabolic parameters at baseline, as well as after 4 and 12 weeks of intervention. Either 500 mg of LLE (50.94 mg of UA) or a placebo was administered to fifty-four healthy adults each day for 12 weeks; no differences in muscle strength, muscle mass, and physical performance were observed between the two groups. However, the right-handgrip strength of female subjects in the LLE group was found to be significantly better than that of subjects in the control group (*P* = 0.047). Further studies are required to determine the optimal dose and duration of LLE supplementation to confirm the first-stage study results for clinical application. ClinicalTrials.gov Identifier is NCT02401113.

## 1. Introduction

Aging is accompanied by a progressive decrease in fat-free mass, mostly due to the loss of muscle mass [1]. In addition, it has been reported that muscle atrophy results from chronic diseases such as diabetes, cancer, congestive heart failure, and chronic obstructive pulmonary disease, along with the disuse condition [2]. This inevitable decline of muscle mass followed by muscle weakness leads to both physical and physiological deteriorations that increase individual's frailty. Sarcopenia, an age-related decrease of muscle mass and strength, has been reported to be associated with physical disability, poor quality of life, high healthcare burden, and mortality [3, 4]. In this context, therapeutic strategies for the improvement of muscle function are important to preserve mobility in later years.

Loquat (*Eriobotrya japonica*) is a plant species belonging to the family Rosaceae and the loquat leaf extract (LLE) has been reported to contain ursolic acid (UA) [5, 6]. UA was found to reduce muscle atrophy and stimulate muscle hypertrophy in vivo study [7]. Moreover, another previous study revealed that the LLE supplements inhibited dexamethasone-induced reduction of muscle strength in Sprague-Dawley rats [8]. Accordingly, those results suggest the possibility of using LLE as a therapeutic agent for the prevention of skeletal muscle atrophy [7]. Based on evidence from these previous studies, we hypothesized that UA, which is the main component of LLE, has a positive effect on skeletal muscle mass, muscle strength, and muscle function in humans.

Therefore, we conducted a randomized, double-blinded, and placebo-controlled trial to investigate the effect of administration of the loquat leaf compound for 12 weeks on skeletal muscle mass, strength, and metabolic muscle markers in healthy adults; the safety of the compound was evaluated as well.

## 2. Material and Methods

### 2.1. Study Subjects and Ethics

The study was approved by the Institutional Review Board at Pusan National University Yangsan Hospital (02-2014-021), and written informed consents were obtained from all subjects prior to the study. Healthy adults were recruited through an official announcement at the tertiary hospital of Yangsan city. The eligibility criteria for enrollment were as follows: age between 19 and 65 years and body mass index (BMI) ranging from 18.5 to 30.0 kg/m^2^.

The exclusion criteria included abnormal liver or renal function (i.e., serum aminotransferase activity > 60 IU/L and serum creatinine concentrations > 1.2 mg/dL); diabetes (diagnosed clinically or fasting glucose level > 126 mg/dL); history of fracture during the previous year; uncontrolled hypertension; history of serious cardiac disease such as angina or myocardial infarction; history of gastrectomy; history of medication for psychiatric disease; administration of oriental medicine including herbs within the past 4 weeks; and evidence of relatively high skeletal mass (more than 110% of the standard lean body mass as measured using the body composition analyzer InBody 720) [9].

### 2.2. Study Design

This study was a randomized, double-blinded, and placebo-controlled trial. A simple randomization technique based on number tables was used to assign each participant to either the intervention or the control group. The participants were assigned randomization numbers sequentially and these randomization codes were held by the company that manufactured the LLE and the dummy placebo. The measurements were conducted by people with no access to the randomization process of the participants.

After the baseline assessment, subjects were randomly allocated to either the LLE- or the placebo-supplemented group. Each subject was instructed to visit the clinic after 4 (±3 days) and 12 (±7 days) weeks from the beginning of the treatment. On each visit, evaluation of muscle mass, anthropometric measurement of body composition, physical performance test, and blood sampling were performed for every participant.

### 2.3. Treatments

The LLE, obtained from dried loquat leaf, contained 102 mg/g of UA as the major component, and each capsule (Korea Pharmacy Corporation, CHO-A PHARM Co., Ltd.) contained 250 mg of LLE. 500 mg (UA 50.94 mg) per day of LLE was administered to the subjects in the intervention group 30 minutes after breakfast and dinner for 12 weeks. The same quantity of placebo was administered to the subjects in the control group twice a day for 12 weeks. The placebo had an identical appearance and taste as the LLE capsule.

### 2.4. Muscle Strength

A Biodex® System 3 Pro isokinetic dynamometer (Biodex, Inc., Shirley, NY, USA) was used to measure the peak torque (TQ) at 60°/s knee extension movements and the peak TQ/body weight was calculated at 60°/s knee extension. The handgrip strength was measured using a hydraulic hand dynamometer (Jamar, Jackson, MI). Three consecutive measurements of handgrip strength of both hands were recorded with subjects sitting in an upward position and their arms at a 90° angle position, and the maximum strength effort was determined.

### 2.5. Body Composition

BMI was calculated as weight (kg) divided by square of height (m^2^). A mercury sphygmomanometer was used to measure the blood pressure of each subject in the sitting position after a 10 min resting period. Two readings, each for systolic and diastolic blood pressure, were recorded at 3 min intervals, and the average of each measurement was used for analyses. A well-trained radiological technologist measured the body composition using a dual-energy X-ray absorptiometry (DXA). We calculated the appendicular skeletal muscle (ASM)/height^2^ and ASM/weight as skeletal muscle mass indices.

### 2.6. Physical Performance

The subject's physical performance was assessed using the short physical performance battery (SPPB), which consists of 3 components: balance, 4 m gait speed, and chair-rise ability [10]. A well-trained study nurse performed all examinations.

### 2.7. Blood Sampling

Blood samples after a 12 h overnight fast were collected at baseline and 4 and 12 weeks after the randomization for general blood testing, biochemical testing, and lipid testing to evaluate metabolic risk factors and monitor on potential adverse effects of LLE. The serum creatinine and blood pyruvate levels were measured by Jaffe's kinetic alkaline picrate method and the enzymatic method, respectively. Lactate concentrations and serum electrolytes levels were measured using an ion-selective electrode, while serum creatinine kinase (CK) concentration was measured using the kinetic ultraviolet method. Liver enzyme and total cholesterol levels were measured with Toshiba TBA200FR (Toshiba Co. Ltd., Tokyo, Japan) using an enzymatic colorimetric method, and low-density lipoprotein (LDL) and high-density lipoprotein (HDL) cholesterol were measured with Toshiba TBA200FR directly. Triglycerides were measured using lipase, glycerol kinase (GK), glycerol-3-phosphate oxidase (GPO), and peroxidase (POD) with a glycerol blank. Fasting blood sugar was measured using a glucose oxidase test method (LX-20, Beckman Coulter, Fullerton, CA, USA), and the serum insulin concentration was measured using Coat-A-Count® Insulin with solid-phase 125I radioimmunoassay.

### 2.8. Nutrition and Exercise Assessments

The diet of each subject was monitored by a semiquantitative food frequency questionnaire at baseline and after 12 weeks. Participants were asked to report the frequency of consumption of 53 food items contained in the semiquantitative food frequency questionnaire over a period of 2 weeks prior to administration [11]. The total intake of energy and essential nutrients including protein, fat, and carbohydrate of participants was calculated using the questionnaire. Physical activity was assessed using the International Physical Activity Questionnaire (IPAQ) at baseline and after 12 weeks [12]. The physical activity levels were expressed as metabolic equivalent- (MET-) minute. METs are multiples of the resting metabolic rates. An MET-minute is computed by multiplying the MET score of an activity by its duration (in minutes). MET-minute scores are equivalent to kilocalories for a person weighing 60 kg.

### 2.9. Statistical Analysis

The primary outcome measure was 60°/s knee extension peak TQ at an angular velocity of 60°/s. The secondary outcome measures included peak TQ/body weight; handgrip strength measurement; and lean mass measured by DXA, SPPB, and other laboratory markers related to the muscle, such as pyruvate, lactate, and creatinine. The average knee extension peak TQ at an angular velocity of 60°/s was set at 67 Nm [13], and the difference in pre- and posttreatment changes in knee peak TQ between the placebo and the control groups was set at 5% with reference to 0–11% from a previous study [14], while the average difference between the placebo and the control groups to be detected was set at 5% of 67 Nm and 3.35 Nm. With a standard deviation (SD) of 3.9 Nm, based on a previous study, a minimum of 21 subjects per group would be required to detect a difference in knee peak TQ (power = 80% and alpha error = 0.05) [15]. Using this calculation, we assumed a 20% dropout rate and selected 27 subjects per group for the present study. When the test data were unavailable, the last recorded data were used in the analysis (called the last observation carried forward). Efficacy analyses were based on the intent-to-treat population of subjects who received at least one dose of the prescribed LLE or placebo. The Shapiro-Wilk test was adopted to test the normality of the variables. Results are expressed as mean ± SD because the variables were all normally distributed. The between-group comparisons for baseline characteristics and the changes after 4 and 12 weeks were performed using a two-sample* t*-test for continuous variables as appropriate or the chi-square test in case of categorical variables. The within-group comparisons were done with a paired* t*-test. *P* value less than 0.05 was considered statistically significant. The SAS ver. 9.3 was used for all statistical analyses.

## 3. Results

### 3.1. Subject

Sixty-five subjects were enrolled; of which, 54 subjects went through the randomization. Twenty-four subjects (88.9%) in the LLE group and 22 subjects (81.5%) in the control group completed the visits. The average compliance rates were 93.2% in the LLE group and 92.5% in the control group; these values did not differ between the groups. The baseline characteristics of the study subjects are presented in Table 1, and no significant differences were observed between the two groups, except in the gender ratio (*P* = 0.004). The number of men in the control group was four times higher than the number in the LLE group, even though they were assigned randomly. Caloric intake, intake of essential nutrients, and physical activity recorded in MET-minute remained unchanged within the group and between the groups throughout the entire 12 weeks.

### 3.2. Muscle Strength

At baseline, the muscle strength parameters did not differ between the two groups (all *P* > 0.05) (Table 2). Throughout the 12 weeks, no significant differences in muscle strength were observed between the two groups (all *P* > 0.05). Statistically, the right-handgrip strength significantly increased in the two groups at 12 weeks (*P* < 0.001, *P* < 0.05) (Table 2). However, no significant differences were reported in the handgrip strength between the two groups over time (Table 2).

**Figure 1:**
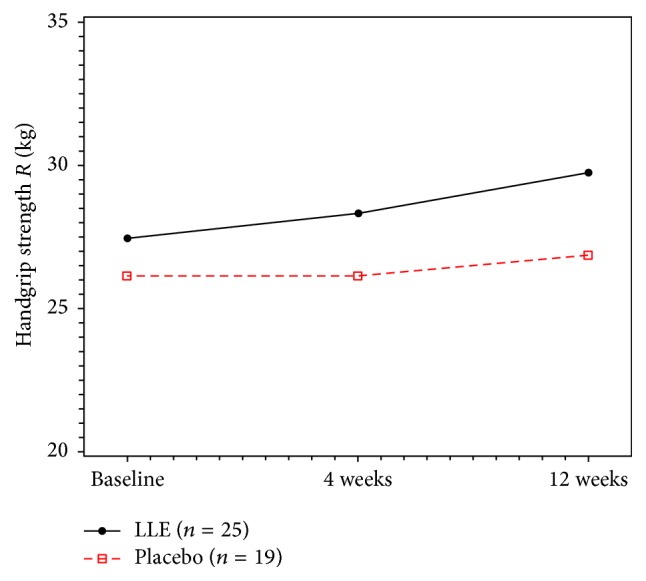
Intention-to-treat analysis on the right-handgrip strength of the female subjects in the LLE and placebo groups. Data are expressed as mean ± SD. Two-sample test between groups for comparison of the difference from baseline (*P* = 0.431, after 4 weeks) (*P* = 0.047, after 12 weeks).

Additionally, we performed the analysis based on gender because the sex proportion differed between the two groups (control group: 8 male subjects [29.6%]; LLE group: 2 male subjects [7.4%]). Statistically significant differences were observed in the right-handgrip strength during the 12 weeks between the LLE group and the control group (Δ right-handgrip strength, 2.3 ± 2.96 versus 0.62 ± 2.28, *P* = 0.047). However, changes during 4 weeks were similar between the two groups (Δ right-handgrip strength, 0.87 ± 2.58 versus 0.26 ± 2.39, *P* = 0.431) (Figure 1).

### 3.3. Muscle Mass

ASM/height^2^ and ASM/weight × 100 showed that the muscle mass had not changed in the two groups at 12 weeks (*P* = 0.924 and *P* = 0.976) (Table 3). After 4 and 12 weeks, the total fat percent decreased in both the groups; however, no significant differences were observed in the two groups (*P* = 0.561 and *P* = 0.125).

### 3.4. Physical Performance

The balance test score SPPB did not change in each group at 12 weeks (*P* = 0.327 and not applicable). However, 4 m gait speed and chair-rising time significantly improved in both groups at 12 weeks (*P* = 0.017 and *P* = 0.033; *P* < 0.001 and *P* < 0.001), with no other differences between the two groups (*P* = 0.699 and *P* = 0.672) (Table 3).

### 3.5. Blood Measurements

No significant differences were observed in the laboratory test results for pyruvate, lactate, electrolyte, glucose, and CK levels and the lipid profile, as well as liver and renal function between the two groups (Table 4).

### 3.6. Safety

One of participants in the control group complained of skin rash and decided to drop out. Otherwise, no other adverse effects were observed in the LLE group.

## 4. Discussion

To our knowledge, the present study is the first randomized, double-blind, and placebo-controlled trial to investigate the efficacy and the safety of LLE supplementation for improving muscle strength, muscle mass, and muscle function. This study also showed that an LLE supplementation dose of 500 mg/day over 12 weeks had no favorable effects on muscle mass and function in healthy adults. However, the right-handgrip strength statistically improved in LLE-supplemented female subjects compared to that in the placebo group female subjects throughout the 12-week period.

Previous studies have suggested therapeutic options for the improvement of muscle mass and function mainly based on a combination of protein supplementation and exercise [16–18]. In a previous study, dietary protein supplementation caused improvements in the physical performance of 65 frail elderly subjects but failed to increase the skeletal muscle mass [16]. In contrast, another study on sarcopenic female patients demonstrated that exercise and simultaneous amino acid supplementation (3 g leucine-rich essential amino acid mixture administered twice a day) enhanced muscle strength and muscle mass variables and physical performances, such as walking speed [17]. In addition, a recent study including 44 healthy young men showed that 3 months of resistance-type exercise training increased skeletal muscle mass, strength, and muscle fiber size. Along with exercise training, a group supplemented daily with dietary proteins (27.5 g) before sleep showed a more demonstrated increase in muscle mass and strength when compared with the group undergoing exercise training alone [18]. Consistent with those results, the Society for Sarcopenia, Cachexia, and Wasting Disease recommended appropriate protein and energy intake with exercise for the prevention of sarcopenia [19].

Several studies suggest new therapeutic strategies for the prevention of aging-associated decline in muscle mass and function [20, 21]. Fish oil-derived n-3 PUFA therapy for 6 months showed significant beneficial effects on thigh volume, handgrip strength, and upper- and lower-body 1-RM muscle strength when compared with the placebo group [20]. In another study, the use of high whey protein-, leucine-, and vitamin D-enriched supplements preserved appendicular muscle mass in obese older adults on a hypocaloric diet and resistance exercise program when compared with that of an isocaloric control group [21]. However, treatments for the prevention and improvement of sarcopenia are still not sufficient to meet the demands.

In oriental medicine, LLE, a common ingredient in teas and food, and UA, a major active component of loquat leaves, are used. A study based on microarray analysis reported that UA reduced skeletal muscle atrophy under two distinct atrophy-inducing stress conditions (fasting and muscle denervation). This particular in vivo study also demonstrated that long-term treatment with UA increased Akt phosphorylation and mRNA expression of insulin-like growth factor-1 [7]. Another study showed that UA directly promoted protein accretion in cultured myotubes but did not modulate myoblast proliferation [22]. However, the risks associated with the use of UA also have to be considered because of unfavorable side effects at high dose, including compromised viability of both myoblasts and myotubes [8] and toxic myopathy [23]. In this context, we performed a human study to evaluate the effect and safety of LLE in improving skeletal muscle mass, strength, and metabolic muscle markers for medical usage.

In the present study, unfortunately, we were unable to show the positive effects of LLE on muscle strength, muscle mass, and muscle function in healthy adults. The negative results could be explained, in part, by the use of insufficient dosage of the active component. Subjects in the intervention group daily received 500 mg of LLE supplement, which contained 50.94 mg of UA. In our preliminary study, we confirmed that 50 mg/kg/day of LLE supplement over a period of 5 weeks increased the muscle mass in rats. Therefore, our planned treatment dose was 1000 mg/day at the beginning of the study. However, we had to decrease the dose to 500 mg/day because a long-term human study using LLE had not been conducted in the past. Secondly, this study excluded exercise training to evaluate primarily the effect of LLE during the study. In a prior study, community-dwelling elderly sarcopenic women were randomly assigned to one of the four groups: exercise and amino acid supplementation (exercise + AAS), exercise, AAS, or health education [17]. The knee extension strength improved significantly only in the exercise + AAS group, and no improvement was seen in the other groups. Lastly, more than 12 weeks may be required to notice positive results. According to the subgroup analysis of our data, the right-handgrip strength of the LLE group significantly increased only after 12 weeks but showed no significant change after 4 weeks.

Our study had some limitations including lack of histological confirmation to determine the action mechanism of LLE in the muscle and inability to measure UA levels in blood. Moreover, despite the randomization process, the female sex ratio was quite high in all the groups. Because testosterone and androgen are known to affect muscle growth and muscle mass, the efficacy of LLE may vary depending on the gender. However, the prospective design of this study compensates in part for these limitations. We stratified the analysis into subjects aged <45 and those aged 45+ owing to the relatively wide age range of study participants; however, the results of subgroup analysis were not different. Our study participants cannot represent entire population because we excluded people with relatively high skeletal muscle mass. Other limitations include insufficient LLE dosage and abstinence from additional exercising for the study's duration. In addition, there was a concern regarding the validity of the physical performance evaluation tool. The SPPB system was adopted for the evaluation of subjects' physical performance score, even though the system was originally intended to be used only in elderly people.

In conclusion, LLE supplementation did not improve muscle strength, muscle mass, and muscle function in healthy adults. However, the right-handgrip strength of female subjects in the LLE group showed significant improvement when compared with that of the female subjects in the placebo group. Further well-designed researches are needed to determine the appropriate dose ranges and duration of LLE supplementation and to increase the usefulness of our first-stage study outcome in various clinical settings.

## Figures and Tables

**Table 1:** Baseline characteristics of the study group.

	LLE (*n* = 27)	Placebo (*n* = 27)	*P* ^(1)^
Age, years	40.0 ± 11.3	38.2 ± 12.0	0.586
Males, %	2 (7.4)	8 (29.6)	**0.004**
Systolic blood pressure, mmHg	113.7 ± 10.7	115.8 ± 12.6	0.509
Diastolic blood pressure, mmHg	73.3 ± 6.8	72.2 ± 11.7	0.671
Non-smoker, %	27 (100)	24 (88.9)	0.721^(2)^
Body mass index, kg/m^2^	22.2 ± 2.4	22.8 ± 2.6	0.410
Activity, METs/week	25.8 ± 24.6	27.3 ± 33.5	0.852
Energy intake, Kcal/day	1709.1 ± 467.2	1816.8 ± 313.9	0.326
Protein intake, g/day	68.8 ± 22.4	70.0 ± 17.2	0.827
Fat intake, g/day	44.9 ± 15.4	45.5 ± 16.0	0.887
Carbohydrate intake, g/day	257.5 ± 70.9	281.8 ± 55.4	0.166

LLE: loquat leaf extract; MET: metabolic equivalent.

One MET is approximately equal to 1 kcal/min for a person weighing 60 kg.

Data are expressed as mean ± SD or in frequency (percent).

^(1)^Statistical differences are based on two-sample *t*-test or chi-square test.

^(2)^Fisher's exact test.

**Table 2:** Comparison of the muscle strength of the two groups.

Variables	Group	Week 0^(1)^	Week 4^(1)^	Δ4-0 weeks	*P* ^(2)^	Week 12^(1)^	Δ12-0 weeks	*P* ^(2)^
60°/s knee extension peak TQ (Rt), Nm	LLE (*n* = 27)	87.8 ± 38.4	89.8 ± 34.3	2.0 ± 16.8	0.543	90.2 ± 33.6	2.4 ± 18.6	0.513
	Placebo (*n* = 27)	101.2 ± 39.5	101.0 ± 33.4	−0.2 ± 20.9	0.969	109.9 ± 45.8	8.7 ± 23.9	0.070
			*P* ^(3)^	0.678		*P* ^(3)^	0.285	

60°/s knee extension peak TQ (Lt), Nm	LLE (*n* = 27)	86.9 ± 31.3	91.5 ± 36.0	4.6 ± 17.5	0.182	92.0 ± 34.5	5.1 ± 15.4	0.094
	Placebo (*n* = 27)	96.9 ± 32.6	104.7 ± 42.0	7.8 ± 19.8	0.051	109.1 ± 41.0	12.2 ± 19.2	**0.003**
			*P* ^(3)^	0.534		*P* ^(3)^	0.143	

60°/s knee extension peak TQ/weight (Rt), Nm/kg	LLE (*n* = 27)	148.8 ± 52.0	153.1 ± 45.7	4.3 ± 28.1	0.435	153.8 ± 45.0	5.1 ± 31.9	0.417
	Placebo (*n* = 27)	161.4 ± 48.4	163.1 ± 44.0	1.7 ± 32.1	0.786	174.6 ± 55.0	13.2 ± 35.2	0.062
			*P* ^(3)^	0.754		*P* ^(3)^	0.376	

60°/s knee extension peak TQ/weight (Lt), Nm/kg	LLE (*n* = 27)	148.9 ± 45.4	156.0 ± 49.1	7.1 ± 27.9	0.196	157.0 ± 46.9	8.1 ± 25.4	0.111
	Placebo (*n* = 27)	155.9 ± 42.0	166.8 ± 50.0	11.0 ± 28.2	0.054	173.4 ± 47.2	17.5 ± 27.9	**0.003**
			*P* ^(3)^	0.617		*P* ^(3)^	0.201	

Handgrip (L), kg	LLE (*n* = 27)	26.8 ± 4.9	27.7 ± 5.9	0.9 ± 2.8	0.100	28.0 ± 6.7	1.2 ± 4.1	0.151
	Placebo (*n* = 27)	30.0 ± 8.8	30.3 ± 9.5	0.3 ± 3.3	0.611	31.3 ± 9.8	1.3 ± 3.7	0.083
			*P* ^(3)^	0.485		*P* ^(3)^	0.908	

Handgrip (R), kg	LLE (*n* = 27)	28.6 ± 6.1	29.4 ± 6.4	0.8 ± 2.6	0.138	31.2 ± 7.2	2.6 ± 3.1	**<0.001**
	Placebo (*n* = 27)	31.1 ± 9.3	31.8 ± 10.1	0.7 ± 4.9	0.436	33.3 ± 11.1	2.2 ± 5.0	**0.031**
			*P* ^(3)^	0.978		*P* ^(3)^	0.748	

Handgrip (mean), kg	LLE (*n* = 27)	27.7 ± 5.4	28.6 ± 5.9	0.9 ± 2.1	**0.045**	29.6 ± 6.7	1.9 ± 3.2	**0.005**
	Placebo (*n* = 27)	30.6 ± 8.9	31.1 ± 9.7	0.5 ± 3.8	0.471	32.3 ± 10.4	1.7 ± 4.0	**0.032**
			*P* ^(3)^	0.714		*P* ^(3)^	0.901	

LLE: loquat leaf extract; TQ: torque; NA: not applicable.

Data are expressed as mean ± SD.

^(1)^ *P* > 0.05 by two-sample test.

^(2)^Paired *t*-test for within-group.

^(3)^Two-sample test between groups for comparison of the difference from baseline.

**Table 3:** Comparison of the muscle mass and muscle function between the two groups.

Variables	Group	Week 0^(1)^	Week 4^(1)^	Δ4-0 weeks	*P* ^(2)^	Week 12^(1)^	Δ12-0 weeks	*P* ^(2)^
ASM/height^2^, kg/m^2^	LLE (*n* = 27)	5.4 ± 0.7	5.6 ± 0.7	0.12 ± 0.21	**0.007 **	5.5 ± 0.7	0.02 ± 0.21	0.662
	Placebo (*n* = 27)	5.9 ± 1.1	6.0 ± 1.2	0.13 ± 0.33	**0.047 **	5.9 ± 1.1	0.02 ± 0.21	0.567
			*P* ^(3)^	0.841		*P* ^(3)^	0.924	

ASM/weight × 100, %	LLE (*n* = 27)	24.7 ± 3.0	25.1 ± 2.9	0.42 ± 0.91	**0.025 **	24.8 ± 3.1	0.06 ± 0.92	0.723
	Placebo (*n* = 27)	25.9 ± 3.8	26.5 ± 4.3	0.57 ± 1.5	0.058	26.0 ± 3.9	0.07 ± 0.91	0.691
			*P* ^(3)^	0.651		*P* ^(3)^	0.976	

Trunk fat percent, %	LLE (*n* = 27)	32.5 ± 5.9	31.1 ± 5.1	−1.41 ± 2.49	**0.007 **	31.3 ± 5.4	−1.24 ± 1.62	**<0.001 **
	Placebo (*n* = 27)	30.7 ± 5.4	29.7 ± 6.0	−1.03 ± 2.31	**0.028 **	30.2 ± 5.2	−0.54 ± 1.7	0.112
			*P* ^(3)^	0.561		*P* ^(3)^	0.125	

Total fat percent, %	LLE (*n* = 27)	34.1 ± 5.0	33.0 ± 4.8	−1.03 ± 1.69	**0.004 **	33.2 ± 4.8	−0.86 ± 1.11	**<0.001**
	Placebo (*n* = 27)	32.1 ± 6.0	31.0 ± 6.5	−1.09 ± 1.78	**0.004 **	31.3 ± 5.9	−0.74 ± 1.29	**0.006 **
			*P* ^(3)^	0.907		*P* ^(3)^	0.736	

SPPB (balance), score	LLE (*n* = 27)	4.0 ± 0.2	4.0 ± 0	0.04 ± 0.19	0.327	4.0 ± 0	0.04 ± 0.19	0.327
	Placebo (*n* = 27)	4.0 ± 0	4.0 ± 0.2	−0.04 ± 0.19	0.327	4.0 ± 0	0 ± 0	NA
			*P* ^(3)^	0.163		*P* ^(3)^	0.322	

SPPB (4 m gait speed), s	LLE (*n* = 27)	2.3 ± 0.3	2.2 ± 0.3	−0.07 ± 0.29	0.197	2.1 ± 0.3	−0.14 ± 0.29	**0.017**
	Placebo (*n* = 27)	2.2 ± 0.3	2.2 ± 0.3	−0.04 ± 0.31	0.537	2.1 ± 0.3	−0.11 ± 0.27	**0.033**
			*P* ^(3)^	0.651		*P* ^(3)^	0.699	

SPPB (chair-rising time), s	LLE (*n* = 27)	10.4 ± 2.6	8.6 ± 1.9	−1.84 ± 2.49	**<0.001 **	7.9 ± 1.7	−2.51 ± 2.23	**<0.001**
	Placebo (*n* = 27)	10.5 ± 3.2	8.3 ± 2.2	−2.21 ± 2.03	**<0.001 **	7.7 ± 2.3	−2.76 ± 2.05	**<0.001 **
			*P* ^(3)^	0.548		*P* ^(3)^	0.672	

ASM: appendicular skeletal muscle; LLE: loquat leaf extract; SPPB: short physical performance battery; NA: not applicable.

Data are expressed as mean ± SD.

^(1)^ *P* > 0.05 by two-sample test.

^(2)^Paired *t*-test for within-group.

^(3)^Two-sample test between groups for comparison of the difference from baseline.

**Table 4:** Comparison of the metabolic parameters between the two groups.

Variables	LLE group (*n* = 27)				Placebo group (*n* = 27)				*P* ^(2)^
	Week 0	Week 12	Δ12-0 weeks	*P* ^(1)^	Week 0	Week 12	Δ12-0 weeks	*P* ^(1)^	
Pyruvate	0.85 ± 0.41	0.75 ± 0.49	−0.11 ± 0.53	0.307	0.87 ± 0.4	0.85 ± 0.31	−0.02 ± 0.38	0.773	0.499
Lactate	1.32 ± 0.89	1.02 ± 0.54	−0.3 ± 0.86	0.085	1.41 ± 0.93	1.31 ± 0.77	−0.11 ± 0.84	0.514	0.419
Creatinine	0.71 ± 0.12	0.66 ± 0.14	−0.06 ± 0.11	**0.017**	0.74 ± 0.18	0.69 ± 0.17	−0.06 ± 0.11	**0.014**	0.990
AST	23.2 ± 4.9	19.8 ± 4.4	−3.4 ± 4.3	**<0.001**	23.6 ± 4.4	20.6 ± 4.0	−3.1 ± 3.5	**<0.001**	0.783
ALT	13.3 ± 4.0	12.5 ± 4.5	−0.7 ± 3.0	0.210	15.5 ± 6.5	13.8 ± 5.1	−1.7 ± 5.3	0.116	0.435
GGT	15.4 ± 7.5	14.3 ± 6.7	−1.2 ± 3.2	0.061	20.3 ± 13.1	18.8 ± 15.2	−1.4 ± 5.0	0.149	0.822
Glucose	89 ± 6.5	91.8 ± 10.0	2.8 ± 8.9	0.113	91.7 ± 9.4	95.0 ± 10.5	3.3 ± 8.1	0.044	0.836
Insulin	4.4 ± 1.5	4.9 ± 2.3	0.5 ± 1.8	0.166	5.4 ± 2.6	5.7 ± 3.3	0.4 ± 3.2	0.561	0.872
CK	88.2 ± 42.4	77.4 ± 29.6	−10.8 ± 40.0	0.173	125.1 ± 195.2	116 ± 137.6	−9.1 ± 68.1	0.493	0.913
Na	140.1 ± 1.5	139.0 ± 2.2	−1.1 ± 2.6	**0.045**	140.2 ± 1.6	139.4 ± 2.0	−0.8 ± 1.9	**0.038**	0.635
K	4.3 ± 0.3	4.2 ± 0.3	−0.1 ± 0.4	0.201	4.4 ± 0.3	4.3 ± 0.2	−0.1 ± 0.3	0.051	0.862
Cl	104.6 ± 1.8	103.6 ± 1.6	−0.9 ± 2.0	**0.022**	104.6 ± 1.8	104.3 ± 2.3	−0.4 ± 1.7	0.272	0.275
HDL	61.3 ± 12.5	60.3 ± 12.6	−1 ± 8.8	0.558	58.9 ± 13.1	58.4 ± 15.0	−0.5 ± 9.7	0.784	0.849
LDL	101.1 ± 25.5	144.3 ± 119.2	43.1 ± 122.3	0.084	100.9 ± 23.1	112.9 ± 28.7	12 ± 21.4	**0.007**	0.199
Triglyceride	80.7 ± 50.8	78.6 ± 35.3	−2.07 ± 31.7	0.736	85.1 ± 46.0	77.7 ± 36.4	−7.4 ± 30.1	0.210	0.526

LLE: loquat leaf extract; AST: aspartate aminotransferase; ALT: alanine aminotransferase; GGT: *γ*-glutamyl transferase; CK: creatinine kinase; HDL: high-density lipoprotein; LDL: low-density lipoprotein.

Data are expressed as mean ± SD.

^(1)^Paired *t*-test for within-group.

^(2)^Two-sample test between groups for comparison of the difference from baseline.
